# Mutations of *OsPLDa1* Increase Lysophospholipid Content and Enhance Cooking and Eating Quality in Rice

**DOI:** 10.3390/plants9030390

**Published:** 2020-03-21

**Authors:** Muhammad Saad Shoaib Khan, Rasbin Basnet, Sulaiman Ahmed, Jinsong Bao, Qingyao Shu

**Affiliations:** 1 National Key Laboratory of Rice Biology and Zhejiang Key Laboratory of Crop Germplasm Resources, College of Agriculture and Biotechnology, Institute of Crop Sciences, Zhejiang University, Hangzhou 310058, China; yusufzai.pathan786@hotmail.com (M.S.S.K.); 11416095@zju.edu.cn (R.B.); 2 Institute of Nuclear Agricultural Sciences, Key Laboratory for Nuclear Agricultural Sciences of Zhejiang Province and Ministry of Agriculture and Rural Affairs, Zhejiang University, Zijingang campus, Hangzhou 310058, China; sulaiman@sippe.ac.cn (S.A.); jsbao@zju.edu.cn (J.B.)

**Keywords:** brown rice, CRISPR, phytic acid, Lysophospholipid, RVA, PLD

## Abstract

Phospholipids belong to a significant class of lipids and comprise ~10% of total lipids in rice grains. Lysophospholipid (LPL) is derived from the hydrolysis of phospholipids and plays an important role in rice grain quality. Our previous study demonstrated that mutations in a phospholipase D gene (*OsPLDα1*) significantly altered lipid metabolites and reduced phytic acid content. In the present study, the effect of two *ospldα1* mutations on LPL and other physicochemical prosperities of brown rice was further investigated, with the aim of assessing the overall importance of *ospldα1* mutations in rice grain quality. Metabolite profiling revealed a ~15% increase in LPL level in both *ospldα1* mutants as compared with their wild-type (WT) parent. Both *ospldα1* mutations significantly lowered the apparent amylose content in brown rice flour (~1.9%) and altered viscosity profiles with significantly increased breakdown (+12.4%) and significantly reduced setback viscosity (−6.2%). Moreover, both *ospldα1* mutations significantly lowered the gelatinization onset, peak temperature and retrogradation percentage of brown rice flour. This study demonstrated that OsPLDα1 plays a crucial role in rice grain quality and its mutation could, in general, improve the cooking and eating quality and nourishment of brown rice.

## 1. Introduction

Phospholipids are the major class of lipids that comprise ~10 % of total grain lipid content [1], and they form complexes with the amylose chain in the endosperm and hence play an essential role in the nutritional and physicochemical properties of rice grain [2]. Phospholipases are phospholipid-hydrolyzing enzymes and are classified into three groups, A, C and D, based on their catalytic activity. Phospholipase A (PLA) hydrolyzes phospholipids to produce lysophospholipids (LPL); Phospholipase C (PLC) cleaves the glycerophosphate bond in phospholipids to generate diacylglycerol (DAG); and Phospholipase D (PLD) degrades phospholipids to yield phosphatidic acid (PA) [3]. PLD has been well-known for its multi-disciplinary functions in plant growth and development [4]. For instance, it plays an important role in pollen tube development [5], seed germination [6], the regulation of abscisic acid (ABA) and jasmonic acid (JA) signaling [7,8] and salt stress response in Arabidopsis [9]. Furthermore, the role of *PLDα1* in seed quality improvement has been recently reported in soybean, where the knockdown of *PLDα* enhances seed quality [10]. Similarly, the silencing of *AtPLDα1* in Arabidopsis improves seed quality and longevity by reducing lipid peroxidation [11].

In comparison to other PLD proteins in rice, OsPLDα1 is considered more analogous to AtPLDα1 [12], suggesting that it could be a good candidate gene for quality improvement in rice grain. LPLs are the derivative of phospholipids in which one or two acyl groups have been removed, and have similar properties to phospholipids [13,14,15]. The LPLs notably influence the eating quality traits of rice, for example, pasting parameters like cool paste viscosity and breakdown are significantly correlated with LPL in specific genotypes [16].

In a recent study, we showed the involvement of *OsPLDα1* in the lipid-dependent phytic acid biosynthesis pathway and its knockout significantly reduced phytic acid content [17]. The same study also revealed that knockout mutations of *OsPLDα1* altered lipid metabolite profiling in rice grain. Tong et al. [18] have demonstrated that the low phytic acid (*lpa*) mutations may disturb the LPL metabolism and modify the apparent amylose content and pasting viscosities in the *lpa* rice mutants. However, whether the *ospldα1* mutations also affected LPL content and the physicochemical properties of brown rice has not been examined. In the present study, rice grains were produced from Xidao #1 and its two *OsPLDα1* knockout mutants in two locations and subjected to an analysis of LPL metabolites and physicochemical properties. Furthermore, the transcriptional level of genes involved in the key steps of phospholipid metabolism was examined to understand the mechanisms leading to the LPL metabolic change.

## 2. Results

### 2.1. Lysophospholipid and Gene Expression Analysis of Ospldα1 Mutants

**Figure 1:**
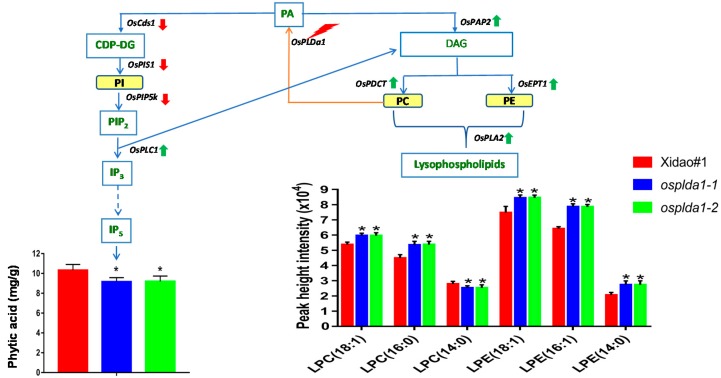
Diagram of biosynthetic pathway of phytic acid and relevant lysophospholipids. LPL: lysophospholipid; PC: phosphatidylcholine; LPC: lysophosphatidylcholine; LPE: lysophosphatidylethanolamine; PE: phosphatidylethanolamine; PI: phosphatidylinositol, PIP_2_: phosphatidylinositol bisphosphate, Ins(1,3,4,5), IP5: phosphatidylinositol pentaphosphate, CDP-DG: cytidine diphosphate-diacylglycerol, IP_3_: inositol 1,4,5 triphosphate, *OsCDS1:* CDP-diacylglycerol synthase 1, *OsPIP5k:* phosphatidylinositol 4-phosphate 5-kinase, *OsPIS1:* phosphatidylinositol synthase 1, *OsPAP2:* phosphatidate phosphatase, *OsPLDa1*: Phospholipase D, *OsPLC1*: Phospholipase C, *OsPLA2*: Phospholipase A2. The red bar designates wild-type, the blue bar indicates mutants *osplda1−1* and the green-colored bar represents *ospldα1–2*. The phytic acid contents were demonstrated in mg/g of dry matter. The lysophospholipid metabolites were obtained from the analysis of three biological samples of brown rice flour and were measured as mean of peak height intensities along with standard deviation. The significant difference between the mutants and the wild-type was shown by asterisk(s) (*p* < 0.05). The thunder bolt represents a knockout mutation; the down- and up-regulated gene expression was indicated by arrows in red and green, respectively.

Lysophospholipids are the main starch lipid in rice grain, while LPE and LPC are the major forms of lysophospholipid in rice, and our study emphasized characterizing the molecular species of these two classes. From the total number of differentially expressed metabolites, six lipid species (total acyl carbon: total acyl double-bond) of LPLs, including LPE (14:0), LPE (16:0), LPE (18:1), LPC (14:0), LPC (16:0) and LPC (18:1), were observed in the wild-type and both *ospldα1* mutants (Table S1). The concentration of the lipid species was calculated by peak height intensity [19], and the results indicated that the detected LPC (18:1), LPC (16:0), LPE (18:1), LPE (16:0) and LPE (14:0) were elevated in both mutants by 11%–32%, respectively. In contrast, we observed a 7% decrease in the LPC (14:0) concentration in both mutants compared to their wild-type parents (Figure 1).

**Figure 2:**
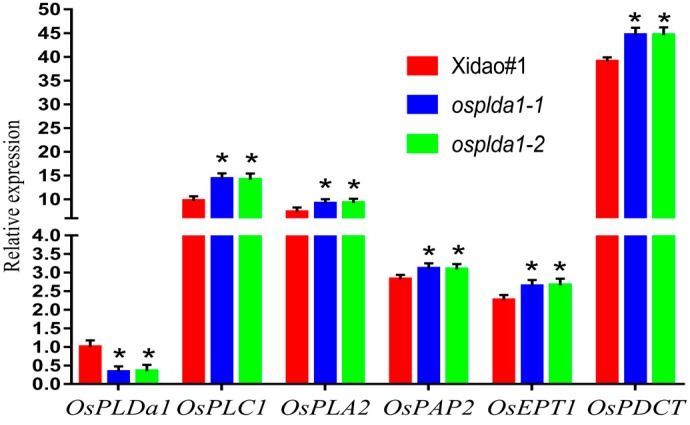
Relative expression level of genes governing the dual metabolic pathway of LPL and phytic acid in rice grains. The expression values of all candidate genes were relative to *OsPLDα1* whereas, *OsActin* was utilized as an internal reference. Data are represented as means with standard deviation, and asterisk(s) represents the significant difference (Duncan’s test, * *p* < 0.05)

The expression of genes encoding phospholipases (*OsPLDα1, OsPLC1, OsPLA2)*, phosphatidate phosphatase (*OsPAP2*), ethanolamine phosphotransferase (*OsEPT1)* and phosphatidylcholine:diacylglycerol cholinephosphotransferase (*OsPDCT)* was significantly different between the wild-type and its mutants. The transcript level of *OsPLDα1* was considerably decreased (67%) in both mutants in comparison to its wild-type. The knock-out mutation in *osplda1* seemed to elevate the expression level of genes working in the LPL pathway. For instance, the transcriptional level of *OsPLC1*, *OsPLA2*, *OsPAP2, OsEPT1* and *OsPDCT* was increased by 48.2%, 25.9%,10.2%, 16.8% and 14.4%, respectively, in the both mutants of *OsPLDα1* (Figure 2).

### 2.2. Apparent Amylose Contents (AAC)

Both amylose and amylopectin play a significant role in determining the starch properties by influencing the starch molecular structure in the pasting and textural characteristics and utilization for processed food. A significant difference was observed in AAC between the wild-type and mutants (*ospldα1-1* and *ospldα1-2*). At Hangzhou, the AAC of the wild-type was 17.17 %, which was higher than that of both mutants *ospldα1-1* (15.33%) and *ospldα1-2* (15.24%) (Table 1). Similarly, at Lingshui, both mutants *ospldα1-1* (15.39%) and *ospldα1-2* (15.40%) exhibited a lower AAC with respect to their wild-type parent.

### 2.3. Thermal and Retrogradation Properties

The gelatinization temperature is a crucial quality index for rice cooking properties. The *ospldα1* mutations seemed to have significantly affected the thermal characteristics of rice flour at both locations (Table 1). At Hangzhou, the onset (T_o_) temperature was found to be lower in *ospldα1-1* (60.71 °C) and *ospldα1-2* (60.91 °C) than the wild-type (Xidao#1, 62.03 °C). There was a significant difference in peak temperature (T_p_), while no significant difference was observed in the conclusion temperature (T_c_) for all samples. The gelatinization enthalpy (ΔH_g_) of mutant lines (*ospldα1-1*; 7.98 j/g_,_ *ospldα1-2*; 7.52 j/g) was significantly higher than that of the wild-type (5.90 j/g). The enthalpy of retrograded starch (ΔH_r_) was also found to be significantly higher in wild-type (0.33 j/g) than the *osplda1-1* (0.16 j/g) and *osplda1-2* (0.17 j/g) mutants. The R% value of the wild-type was almost three times greater than those of mutant lines. The results of thermal and retrogradation properties of rice samples harvested at Lingshui were consistent with those at Hangzhou (Table 1), demonstrating that the differences between the wild-type and mutants were largely caused by the *ospldα1* mutations.

### 2.4. Concentration of Phytic Acid in Mutants

At Lingshui, the *ospldα1-1* and *ospldα1-2* both had a 10.85% reduction in phytic acid contents (Figure S1), while an 8.28% decrease in total phosphorus contents with respect to their wild-type was observed (Figure S1). Thus, this represents that *ospldα1* mutant lines demonstrated a consistent performance with our previous experiment in Hangzhou, where these mutant lines exhibited a 9.9% reduction in phytic acid content [17].

### 2.5. Pasting Properties

**Figure 3:**
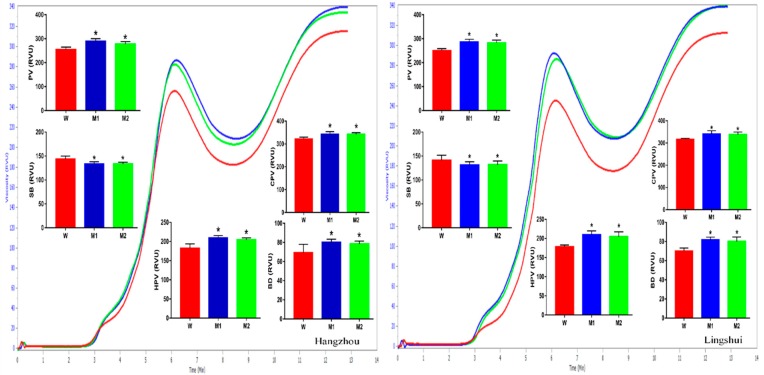
Pasting properties of brown rice of *ospldα1-1*(M1) and *ospldα1-2* (M2) mutants along with their wild-type Xidao#1 (W) harvested from plants grown at Hangzhou, Zhejiang and Lingshui, Hainan. The displayed data comprises the mean of three biological repeats and data significantly different from the wild type are shown by asterisk(s) (Duncan’s test, * *p* < 0.05).

Pasting is an important physicochemical characteristic of starch, relating to the eating and cooking quality of rice. The pasting properties of *ospldα1-1* and *ospldα1-2* brown rice flour differed significantly from the wild-type. The RVA results were consistent in both mutants grown at Hangzhou and Lingshui (Figure 3). At both locations, the *ospldα1* mutants had an overall increase in peak viscosity (PV) by 14.4%, hot paste viscosity (HPV) by 17.6%, breakdown (BD) by 12.4%, and cold paste viscosity (CPV) by 7.8%, while the setback (SB) viscosity was reduced by 6.2%, with respect to the wild-type.

## 3. Discussion

### 3.1. Association of Lysophospholipid with Phytic acid in Osplda1 Brown Rice Flour

At present, the consumption of brown rice grains is increasing because of its health benefits [20]. However, the presence of an anti-nutrient agent (phytic acid) and poor eating quality makes it hard for the consumers to accept brown rice as a substitute for white rice. Keeping this in mind, previously we analyzed the mutational impact of *OsPLDα1* in brown rice grains and observed about a 10% reduction in the phytic acid content in mutants through the lipid-dependent phytic acid biosynthetic pathway [17]. In the current report, we reconfirmed the lower accumulation of phytic acid in the grains of *ospldα1* mutant plants grown together with their parent at a faraway location (Lingshui). Furthermore, we proceeded with an investigation into the lyso form of phospholipids (lysophospholipids), because they are known to play an essential role in the eating and cooking quality of rice. Tong et al. [18] revealed that the LPL level was changed in *lpa* mutants, but the increase or decrease was dependent on causative genes. However, the connection between the phytic acid and LPL biosynthetic pathway has not been fully understood to date. The mutations of *OsPLDα1* not only caused a reduction in phytic acid, but also altered the phosphatidic acid metabolites [17].

We hypothesized that the reduction in the phytic acid and change in the phospholipid metabolites resulting from *OsPLDα1* knockout might also alter the profile of LPL metabolites. The phosphatidic acid is the common contributor to both the lipid-dependent phytic acid and the LPL biosynthetic pathway (Figure 1). There are several pathways for the production of phosphatidic acid, among those the production of phosphatidic acid from phosphotidylcholine hydrolysis, catalyzed by PLD enzyme, is considered to be the most imperative. In the present study, we demonstrated that the mutation of *OsPLDα1* led to an overall increase in LPL content in brown rice, most probably through the acylation of diacylglycerol (DAG), which yields PC and PE [21,22]. There are two pathways for the generation of DAG, i.e., PLD-derived phosphatidic acid catalyzed by phosphatidate phosphatase (encoded by *OsPAP2*) and the hydrolysis of Phosphatidylinositol 4,5-biphosphate by phospholipase C (encoded by *OsPLC1*). In our previous study, we had observed a down-regulation of phosphatidylinositol 4-phosphate 5-kinase (*OsPIP5k*), phosphatidylinositol synthase 1 (*OsPIS1*) and CDP-diacylglycerol synthase 1 (*OsCDS1*) genes at the upstream of the phospholipid-dependent phytic acid pathway [17]. In the *osplda1* mutants, we observed more transcriptional elevation of *OsPLC1* than *OsPAP2,* through which DAG biosynthesis might be up-regulated. The results of the present study are consistent with a previous study, which suggested that PA has a minor contribution to the production of DAG compared to PLC [23]. This indicates that *OsPLC1* has a major involvement in the generation of DAG, as compared to the PLD-derived phosphatidic acid pathway.

The higher activity of PLA2 could contribute to the increase in LPL content in *Arabidopsis* [24]. As phospholipase Dα1 was attenuated in both *ospldα1* mutants, LPC and LPE are more likely to be hydrolyzed from PC and PE by OsPLA2, which is supported by the greater transcription of *OsPLA2* in *ospldα1-1* and *ospldα1-2* (Figure 2). Moreover, our results are consistent with a previous study, in which *lpa* mutant lines derived from a cross of KBNT-*lpa* and Jiahe 218 exhibited an increase in LPC (18:1), LPC (16:0), LPE (18:1), LPE (16:0) and LPE (14:0), and a decrease in LPC (14:0) concentration [25]. Previously it has been reported that LPC 16:0 and LPC 18:1 were considerably associated with *OsPLD 1* and *OsPLA_2_*, respectively [2]. This supports our finding, as the metabolite profiling demonstrated a higher level of LPC 16:0 and LPC 18:1 in *osplda1* mutants, implying a negative association of *OsPLDa1* with LPL contents.

### 3.2. Ospldα1 Mutations Significantly Affected the Cooking and Eating Properties of Brown Rice

Rice grain quality is a multidimensional attribute consisting of various factors like amylose content, gelatinization temperature and starch-pasting viscosity [25]. LPL contents were reported to be positively correlated with amylose contents [26] and might contribute to the pasting as well as the thermal properties of nonwaxy rice starch [27]. Here, both of the *osplda1* mutant lines showed lower AAC than the wild-type, while the LPL contents were higher in the mutants. These results are consistent with a previous report where *lpa* lines from KBNT-*lpa* exhibited higher LPL contents but lower amylose contents [18]. This indicates that, in certain *lpa* rice mutants, amylose contents are negatively correlated with LPL contents. LPL significantly affects the rice thermal and starch-pasting properties [28]. In brown rice flour of *osplda1* mutant lines, a significant upsurge in pasting parameters with the increase in LPL indicates that the elevation of LPL components improves the palatability of cooked rice by enhancing the pasting properties. In another study, it was stated that, in white rice flour, the LPL had a negative correlation with BD [27]; this difference might happen because of the use of brown rice flour in this experiment.

Different parameters of RVA represent diverse characteristics of cooking rice. For instance, the higher setback (SB) and smaller breakdown (BD) value indicates a higher hardness of cooked rice. On the contrary, a larger BD and smaller SB indicated the softening and stickiness of cooked rice [29]. In this study, both *ospldα1* mutants showed a higher peak viscosity (PV) and BD and a lower SB than their parent Xidao#1; this indicates that the cooked brown rice of *ospldα1* mutants is likely to be much softer than its wild-type parent. This is also supported by its AAC value, which has a generally positive correlation with SB and negative correlation with BD [30]. The mutants have much lower AAC compared to their parent, indicating that a softer texture could be expected in the brown rice. Furthermore, the lower gelatinization temperature (To and Tp) in mutant brown rice suggests that a lower energy input is required to cook *ospldα1* mutants with comparison to their parent. Similarly, a lower retrogradation trend (low ΔHr) and percentage (%R) of mutants compared to the parent indicated that the cold brown rice could maintain a much softer texture than the parent. All these results suggest a better culinary and eating quality for the brown rice of the mutants.

## 4. Materials and Methods

### 4.1. Sample Materials

Two knockout phospholipase Dα1 (Os01g0172400) mutant lines, *osplda1-1* and *osplda1-2* were previously developed by CRISPR/Cas9 targeted mutagenesis of a *japonica* rice cultivar (Xidao #1) [17]. Both mutants alongside with their parent were cultivated in three replication plots of 6 × 8 plants, from April to October, 2017, at the paddy fields of Zhejiang seed Co. in Hangzhou during rice-growing season, and, during December 2017 to March 2018 at the winter breeding station of Zhejiang University in Lingshi, Hainan Province. Rice grains harvested from both locations were used for analyzing physicochemical and thermal properties. Rice grains were dehulled and ground by cyclone mill with a 0.5 mm sieve (UDY Corporation, Fort Collins, Colorado, USA). The brown rice flour samples were freeze-dried for 48 h and stored at −18 °C until further analysis.

### 4.2. Metabolite Profiling

The acyl group of lysophosphatidylethanolamine (LPE) and lysophosphatidylcholine (LPC) were noted as acyl cation and anion from suitable negative and positive precursors, respectively. The sub-molecular lipid classes of LPC and LPE were evaluated by [M + H] ^+^ and [M − H]^−^ adduct ions, respectively. The total differential metabolite profile of plants harvested in Hangzhou [17] was utilized to dig out the LPL components.

### 4.3. Apparent Amylose Content (AAC)

The brown rice flour (10 mg) was transferred to 10 mL glass tube with tap, added 100 µL ethanol (95%) and mixed gently. Then, 900 µL of sodium hydroxide (NaOH) was added; after that, samples were placed on boiling water for 10 min until the solution became clear and lump free. The samples were cooled at room temperature and then diluted by adding 9 mL distilled water. A total of 200 µL of dilution was pipetted into a 5 mL tube and added to a 3.8 mL I_2_ mixture solution (1.5 mL 0.2% I2-KI + 1M acetic acid + 97.5 mL distilled water). The final solution was cooled down at room temperature and optical density (OD) was measured at 620 nm. The standard curve was prepared concurrently by utilizing rice flour samples with known AAC (1.5%, 10.4%, 16.2%, 26.5%) to analyze the AAC of the individual sample.

### 4.4. RVA Analyses

The pasting properties of brown rice were evaluated by employing Rapid Visco Analyser (RVA, Model 3D; Newport Scientific, Warriewood, NSW, Australia) with the Thermocline for Windows software (version 1.2). The flour (3g, 12% moisture basis) samples were homogenized in 25 g distilled water. The “standard 1” program was used; the temperature profile is as follows: 50 °C temperature for 1 min and subsequently raised at 12 °C min^−1^ to 95 °C, then further held at the same temperature for 2.5 min, and then decreased at 12 °C min^−1^ to 50 °C, and held for 2 min. The viscosity was presented as Rapid Visco Unit (RVU). The parameters for pasting included PV, HPV, CPV, BD, and SB and were derived from the software.

### 4.5. Thermal and Retrogradation Properties

Differential scanning calorimeter Q20 (TA Instruments, New Castle, DE) was used to determine thermal and retrogradation properties. Brown rice flour (2.0 mg, dry basis) was weighed in aluminum pan, followed by adding 6 µL distilled water, and hermetically sealed. The samples were equilibrated for 2 h at room temperature and then heated over the range of 30–110 °C with a rate of 10 °C/min. The DSC parameters such as onset temperature (T_o_), peak temperature (T_p_), conclusion temperature (T_c_), enthalpy of gelatinization (ΔH_g_) were recorded by universal Analysis 2000 (version 4.4A). The sample pans were kept in the refrigerator for 7 days at 4 °C. The rescanning of sample pans was done from 30 to 110 °C at rate of 10 °C/min and the enthalpy of retrograde (ΔH_r_)_)_ and percentage of retrogradation (R% = ΔH_r_/ ΔH_g_ × 100) was calculated [31].

### 4.6. qRT-PCR Assay

Developing seeds harvested from Lingshui were used for total RNA extraction. The relative gene expression analysis was done according to methods described by [17]. The sequences of the specific primer for each gene are mentioned in the Table S2.

### 4.7. Evaluation of Phytic Acid Content

Phytic acid contents were determined from brown rice flour (1 g) by employing the Megazyme phytic acid assay kit (Megazyme International, Ireland) and absorbance was detected at 655 nm by utilizing MWGT Sirus HT- TRF Spectrophotometer (Biotek, Winooski, VT, USA), as previously described by [32].

### 4.8. Statistical Analysis

All statistical analysis was performed by suing SPSS 20.0 software. The data are presented as mean ± standard deviation (SD) based on three biological repeats. 

## 5. Conclusions

In this report, we demonstrated that the knockout of *OsPLDα1* leads to the transcriptional elevation of phospholipase C- and phospholipase A2-encoding genes, which resulted in a higher accumulation of LPL in *ospldα1* mutants. The increase in LPL components seems to significantly modify the pasting (PV, HPV, BD, CPV) and thermal (ΔH_g_) properties in *ospldα1* mutants. This suggests that the cooked rice of *ospldα1* mutants would be much softer than their wild-type parent. Hence, *OsPLDα1* plays a significant role in improving rice grain quality through the alteration of the metabolites of LPL and reduction in phytic acid contents via the LPL biosynthetic pathway and phytic acid biosynthesis pathway, respectively. The deployment of *ospldα1* mutant in rice breeding programs could enable the development of rice with improved eating and cooking qualities.

## Figures and Tables

**Table 1:** Apparent amylose content, gelatinization and retrogradation properties of brown rice flour obtained from grains of plants grown at two locations.

	Locations	AAC (%)	T_o_ (°C)	T_p_ (°C)	T_c_ (°C)	ΔH_g_ (j/g)	ΔH_r_ (j/g)	R%
Xidao#1	Hangzhou	17.17 ± 0.13 ^a^	62.03 ± 0.27 ^a^	71.38 ± 0.47 ^a^	77.95 ± 0.55 ^a^	5.75 ± 0.41 ^a^	0.33 ± 0.09 ^a^	5.91 ± 1.13 ^a^
*osplda1-1*		15.33 ± 0.42 ^b^	60.71 ± 0.34 ^b^	70.96 ± 0.15 ^b^	77.22 ± 0.11 ^a^	7.98 ± 0.87 ^b^	0.16 ± 0.01 ^b^	2.11 ± 0.09 ^b^
*osplda1-2*		15.24 ± 0.15^b^	60.91 ± 0.33 ^b^	70.41 ± 0.20 ^b^	77.45 ± 0.65 ^a^	7.65 ± 0.51 ^b^	0.17 ± 0.01 ^b^	2.3 ± 0.11 ^b^
Xidao#1	Lingshui	17.28 ± 0.11^a^	61.81 ± 0.21 ^a^	71.07 ± 0.09 ^a^	77.90 ± 0.48 ^a^	5.64 ± 0.28 ^a^	0.36 ± 0.01 ^a^	6.51 ± 0.38 ^a^
*osplda1-1*		15.39 ± 0.39^b^	60.60 ± 0.17 ^b^	70.33 ± 0.40 ^b^	77.40 ± 0.31 ^a^	7.61 ± 0.48 ^b^	0.17 ± 0.01 ^b^	2.2 ± 0.17 ^b^
*osplda1-2*		15.4 ± 0.18^b^	60.68 ± 0.13 ^b^	70.47 ± 0.38 ^b^	77.21 ± 0.29 ^a^	7.72 ± 0.48 ^b^	0.17 ± 0.03 ^b^	2.3 ± 0.16 ^b^

Duncan’s multiple range tests was used to determine significant difference (*p* < 0.05) and it was represented as different letters (a and b) within the same column. R%, percent of retrogradation; ΔHr, enthalpy of retrograde rice flour; ΔHg, enthalpy of gelatinization; To, onset temperature; Tc, conclusion temperature; Tp, peak temperature; AAC, apparent amylose content.

## Supplementary Materials

The following are available online at https://www.mdpi.com/2223-7747/9/3/390/s1, Figure S1: Total phosphorus (total P) and phytic acid contents in brown rice harvested from plants grown at Lingshui, Hainan, Table S1: Differentially expressed Lysophospholipids in wild-type and mutant rice seeds, Table S2: Primers for QPCR analysis.
