# Supplementary material for: Mutations of OsPLDa1 Increase Lysophospholipid Content and Enhance Cooking and Eating Quality in Rice
Source: Plants (Basel). 2020 Mar 21;9(3):390. doi: 10.3390/plants9030390 (PMC7154823; doi:10.3390/plants9030390)
Supplement: Supplementary file 1 [file plants-09-00390-s001.pdf]

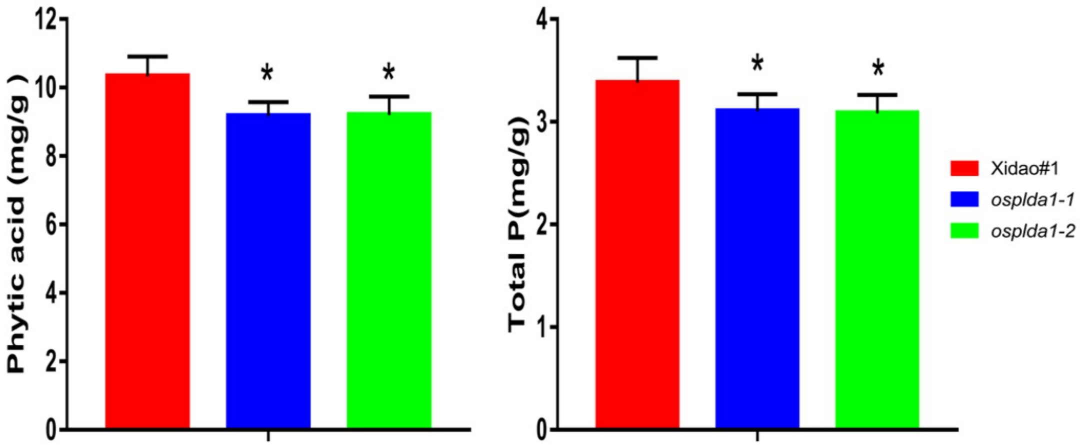

Figure S1. Total phosphorus (total P) and phytic acid contents in brown rice harvested from plants grown at Lingshui, Hainan. Data are the mean of three biological repeats. Error bars represent standard deviation. Data with an asterisk(s) are significantly different from wild type (Duncan's test, * $P < 0.05$).

Table S1. Differentially expressed Lysophospholipids in wild-type and mutant rice seeds.

Gen.	OMI.	RT.	Formula	Adducts	ID	Lipid Class	PHI.	T.T
X1	560.31	1.31	C26H52NO7P	M+K	LMGP01050138	LPC(18:1)	55527.46	0.03
X1	560.31	1.31	C26H52NO7P	M+K	LMGP01050029	LPC(18:1)	53469.16	0.03
X1	560.31	1.31	C26H52NO7P	M+K	LMGP01050080	LPC(18:1)	52310.36	0.03
X1	518.3	1.10	C24H50NO6P	M+K	LMGP01070006	LPC(16:0)	55891.42	0.05
X1	518.3	1.10	C24H50NO6P	M+K	LMGP01070006	LPC(16:0)	55299.38	0.05
X1	518.3	1.10	C24H50NO6P	M+K	LMGP01070006	LPC(16:0)	53361.89	0.05
X1	506.26	0.98	C22H46KNO7P	M+K	LMGP01050012	LPC(14:0)	28282.94	0.03
X1	506.26	0.98	C22H46KNO7P	M+K	LMGP01020009	LPC(14:0)	28286.39	0.03
X1	506.26	0.98	C22H46KNO7P	M+K	LMGP01050073	LPC(14:0)	27017.38	0.03
M1-1	560.31	1.31	C26H52NO7P	M+K	LMGP01050138	LPC(18:1)	59916.89	0.03
M1-1	560.31	1.31	C26H52NO7P	M+K	LMGP01050029	LPC(18:1)	58085.49	0.03
M1-1	560.31	1.31	C26H52NO7P	M+K	LMGP01050080	LPC(18:1)	61088.15	0.03
M1-1	518.3	1.10	C24H50NO6P	M+K	LMGP01070006	LPC(16:0)	43704.22	0.02
M1-1	518.3	1.10	C24H50NO6P	M+K	LMGP01070006	LPC(16:0)	42033.29	0.02
M1-1	518.3	1.10	C24H50NO6P	M+K	LMGP01070006	LPC(16:0)	44832.18	0.02
M1-1	506.26	0.98	C22H46KNO7P	M+K	LMGP01050012	LPC(14:0)	25076.14	0.03
M1-1	506.26	0.98	C22H46KNO7P	M+K	LMGP01020009	LPC(14:0)	25861.8	0.03
M1-1	506.26	0.98	C22H46KNO7P	M+K	LMGP01050073	LPC(14:0)	24935.91	0.03
M1-2	560.31	1.31	C26H52NO7P	M+K	LMGP01050138	LPC(18:1)	60642.04	0.03
M1-2	560.31	1.31	C26H52NO7P	M+K	LMGP01050029	LPC(18:1)	59412.83	0.03
M1-2	560.31	1.31	C26H52NO7P	M+K	LMGP01050080	LPC(18:1)	58852.29	0.03
M1-2	518.3	1.10	C24H50NO6P	M+K	LMGP01070006	LPC(16:0)	43784.39	0.02
M1-2	518.3	1.10	C24H50NO6P	M+K	LMGP01070006	LPC(16:0)	43902.42	0.02
M1-2	518.3	1.10	C24H50NO6P	M+K	LMGP01070006	LPC(16:0)	43573.99	0.02
M1-2	506.26	0.98	C22H46KNO7P	M+K	LMGP01050012	LPC(14:0)	24879.69	0.03
M1-2	506.26	0.98	C22H46KNO7P	M+K	LMGP01020009	LPC(14:0)	25694.23	0.03
M1-2	506.26	0.98	C22H46KNO7P	M+K	LMGP01050073	LPC(14:0)	25394.21	0.03
X1	452.27	0.95	C21H44NO7P	M-H	LMGP02050002	LPE(16:0)	64521.47	0.03
X1	452.27	0.95	C21H44NO7P	M-H	LMGP02050036	LPE(16:0)	64159.24	0.03
X1	452.27	0.95	C21H44NO7P	M-H	LMGP02050002	LPE(16:0)	63890.41	0.03
X1	478.29	1.30	C23H46NO7P	M-H	LMGP02050064	LPE(18:0)	72410.26	0.01
X1	478.29	1.30	C23H46NO7P	M-H	LMGP02050039	LPE(18:0)	72329.13	0.01
X1	478.29	1.30	C23H46NO7P	M-H	LMGP02050004	LPE(18:0)	79469.16	0.01
X1	424.24	1.0	C19H40NO7P	M-H	LMGP02050033	LPE(14:0)	19786.42	0.04
X1	424.24	1.0	C19H40NO7P	M-H	LMGP02050003	LPE(14:0)	20972.25	0.04
X1	424.24	1.0	C19H40NO7P	M-H	LMGP02050033	LPE(14:0)	21057.28	0.04
M1-1	452.27	0.95	C21H44NO7P	M-H	LMGP02050002	LPE(16:0)	79784.51	0.03
M1-1	452.27	0.95	C21H44NO7P	M-H	LMGP02050036	LPE(16:0)	77113.08	0.03
M1-1	452.27	0.95	C21H44NO7P	M-H	LMGP02050002	LPE(16:0)	79022.22	0.03
M1-1	478.29	1.30	C23H46NO7P	M-H	LMGP02050064	LPE(18:0)	88906.87	0.02
M1-1	478.29	1.30	C23H46NO7P	M-H	LMGP02050039	LPE(18:0)	86185.09	0.02

M1-1	478.29	1.30	C23H46NO7P	M-H	LMGP02050004	LPE(18:0)	87088.37	0.02
M1-1	424.24	1.0	C19H40NO7P	M-H	LMGP02050033	LPE(14:0)	27166.23	0.02
M1-1	424.24	1.0	C19H40NO7P	M-H	LMGP02050003	LPE(14:0)	27981.68	0.02
M1-1	424.24	1.0	C19H40NO7P	M-H	LMGP02050033	LPE(14:0)	26855.73	0.02
M1-2	452.27	0.95	C21H44NO7P	M-H	LMGP02050002	LPE(16:0)	78484.14	0.01
M1-2	452.27	0.95	C21H44NO7P	M-H	LMGP02050036	LPE(16:0)	79201.06	0.01
M1-2	452.27	0.95	C21H44NO7P	M-H	LMGP02050002	LPE(16:0)	77993.84	0.01
M1-2	478.29	1.30	C23H46NO7P	M-H	LMGP02050064	LPE(18:0)	87642.17	0.04
M1-2	478.29	1.30	C23H46NO7P	M-H	LMGP02050039	LPE(18:0)	86791.83	0.04
M1-2	478.29	1.30	C23H46NO7P	M-H	LMGP02050004	LPE(18:0)	88268.99	0.04
M1-2	424.24	1.0	C19H40NO7P	M-H	LMGP02050033	LPE(14:0)	26858.17	0.02
M1-2	424.24	1.0	C19H40NO7P	M-H	LMGP02050003	LPE(14:0)	28012.49	0.02
M1-2	424.24	1.0	C19H40NO7P	M-H	LMGP02050033	LPE(14:0)	27152.32	0.02

Gen: Genotypes, X1: Xidao#1, M1-1: Ospldα1-1mutant, M1-2: Ospldα1-2, wild-type OMI: Observed m/z of molecular ion, RT: Retention time, ID: Lipid ID of identified compound, PHI: Peak height intensity, T.T: T-test p-value.

Table S2. Primers for QPCR analysis

Sn	Primer ID	Description	Primer Sequence (5'-3')
1	OsPLDα1-F	Phospholipase D α1	TGGGTAACCGTGAGGTGAAGCAG
	OsPLDα1-R		CCATGGCGATCTCAGAGTCCCTAG
2	OsPLC1-F	Phospholipase C	TGAGTACTCCACCCACCACA
	OsPLC1-R		CAGCTCATCATCAAGCCAACA
3	OsPLA2-F	Phospholipase A2	CCTCCATCATCTCACCGGG
	OsPLA2-R		TAGGGAGGAAGGGAGATCGAG
4	OsPAP2-F	Phosphatidate phosphatase	CAACCCTGGTCCCGAGCTA
	OsPAP2-R		GAGTCGATTCAGGCGGACG
5	OsEPT1-F	Ethanolaminephosphotransferase	GTTGGCCGGAGTTCTTGTCT
	OsEPT1-R		TAGCACAAGGCCTTACCACA
6	OsPDCT-F	Phosphatidylcholine:diacylglycerol cholinephosphotransferase 1	TGCCGTAAAGCTACGGTCAAT
	OsPDCT-R		CATCAGTTTCGCGTGTGCTC
